# Advancing feminist innovation in sport studies: A transdisciplinary dialogue on gender, health and wellbeing

**DOI:** 10.3389/fspor.2022.1060851

**Published:** 2023-01-04

**Authors:** Holly Thorpe, Sheree Bekker, Simone Fullagar, Nonhlanhla Mkumbuzi, Sophia Nimphius, Madeleine Pape, Stacy T. Sims, A. Travers

**Affiliations:** ^1^ School of Health, University of Waikato, Hamilton, New Zealand; ^2^ Department for Health, University of Bath, Bath, United Kingdom; ^3^ Department of Tourism, Sport and Hotel Management, Griffith University, Nathan, AU-QLD, Australia; ^4^ Department of Physiology, Midlands State University, Gweru, Zimbabwe; ^5^ School of Medical and Health Sciences, Edith Cowan University, Joondalup, AU-WA, Australia; ^6^ Institute of Sports Science, Université de Lausanne, Lausanne, Switzerland; ^7^ AUT Sports Performance Research Institute, Auckland University of Technology, Auckland, New Zealand; ^8^ Department of Sociology and Anthropology, Simon Fraser University, Burnaby, BC, Canada

**Keywords:** female athlete health, gender, transdisciplinary, health and wellbeing, feminist sport science

## Abstract

Athlete health and wellbeing requires a holistic, multidimensional approach to understanding, supporting, and treating individual athletes. Building more supportive, inclusive, and equitable environments for the health and wellbeing of women and gender expansive people further requires gender-responsive approaches that promote broader cultural change. Feminist sport and exercise medicine practitioners, sports scientists, and social science researchers are increasingly coming together in their efforts to do this work. However, working across disciplines inevitably includes an array of ontological, epistemological, and political challenges. In this paper, we offer a curated ‘dialogue’ with a group of feminist scholars engaged in research and practice across disciplines, bringing them together to discuss some of the most pressing gendered issues in sport today (i.e., ACL injury, concussion, menstruation in sport, mental health, gender categories). In so doing, we amplify the voices of those working (empirically and clinically) at the disciplinary intersections of gender, sport and health, and learn about some of the current and future possibilities for transdisciplinary innovations and strategies for building (responsiveness to) cultural change.

## Graphical abstract

GRAPHICAL ABSTRACT. 
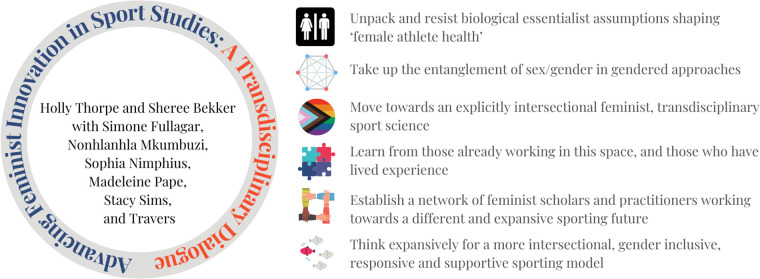

## Introduction

After decades of women's and gender expansive people's health and wellbeing in sport being overlooked, ignored or silenced in sport science and medicine (1–4), we are arguably in a new era. Sportswomen are increasingly the focus of research led by and for women in sport. Many (not all) international and national sports organisations are finally prioritising and investing in more gender-responsive policies, practices and research. Research focused on sports women's health and performance is increasingly being funded and published. While such signs of change are hopeful, it is important to acknowledge the impetus for such investments. The recent investment in so-called ‘female athlete health’ and movement towards systemic changes that recognize and respect the diversity of gendered experiences in sport, are the result of courageous athletes speaking out about their experiences of patriarchal and heteronormative systems that have compromised their immediate and longterm health, wellbeing and performances (5, 6). Furthermore, researchers, practitioners, and activists have been calling for greater understanding, awareness, education, accountability and cultural change for decades. While we are optimistic that *some* sports organisations are willing to revisit and revise structures that have caused so much damage to previous generations of women and gender diverse athletes, this is not to take our eye off the game and to embark upon uncritical celebration. With women's sport, and ‘female athlete health’, in the spotlight, this is a critical time to take stock of how we've come to this position, and the challenges that lay ahead as women's sport becomes a priority area for policy makers, scientists, leaders, coaches and managers.

The authors and contributors to this paper are diverse in that we come from different disciplinary, sporting, gender and national backgrounds and experiences. Despite our differences, we come together in our understanding of the complexities of athlete health and wellbeing, and the need for more holistic, multidimensional approaches to understanding, supporting, and treating individual athletes. Building more supportive, inclusive, and equitable environments for the health and wellbeing of women and gender expansive people further requires gender-responsive approaches that promote broader cultural change. Feminist sport and exercise medicine practitioners, sports scientists, and social science researchers are increasingly coming together in their efforts to engage in inter- and transdisciplinary work on a range of topics, including gendered approaches to sports injury (7), menstrual health (8), Relative Energy Deficiency in Sport (9–11) and research design in exercise and sport science (12, 13).

Yet the ways of working across the disciplines varies widely across gender, sport, health and wellbeing scholarship and practice, and thus it is necessary here to comment briefly on what we see as the key differences between inter- and transdisciplinary research. Whereas interdisciplinarity refers to research in which “an issue is approached from a range of disciplinary perspectives and integrated to provide a systematic outcome” (14), *p*. 400), transdisciplinarity can be understood as a *dialogue* with scholars from the social and physical sciences “in the interests of better addressing substantive interests that are of shared concern” (15), *p*. 265).

Despite claims of and calls for transdisciplinary research in gender, health and wellbeing in sport, working across disciplines inevitably includes an array of ontological, epistemological and political challenges. Such work can be particularly challenging for feminist scholars with different understandings of the workings of power and knowledge, and varied perspectives on strategies for change (16–18). In this paper, we offer a curated ‘dialogue’ with a group of feminist sport scholars each of whom are involved in projects working across disciplines in a range of ways (ranging from interdisciplinarity to transdisciplinarity), coming together to discuss some of the most pressing gendered issues in sport today. In so doing, the cross-disciplinary dialogue of this paper is intended to give rise to new innovations that move beyond any one discipline-specific approach. In fact, having this dialogue is an important initial stage, necessary to move into a new era of transdisciplinary development of new models, concepts and strategies to improving athlete health and wellbeing.

As two feminist sport scholars who have worked across the disciplines and with a range of sports organizations in our efforts towards building gender inclusive sporting environments, we (Holly and Sheree) recognize the practical, logistical and emotional challenges of this work. Holly lives in Aotearoa New Zealand and is a feminist sociologist with a focus on the critical role of sporting cultures on athlete health and wellbeing, and an inaugural member of the High Performance Sport New Zealand transdisciplinary working group WHISPA (Healthy Women in Sport: A Performance Advantage). Through her work with WHISPA, she has organized three national symposiums focused on women's health and wellbeing in sport, collaborated with sports doctors, physiologists, psychologists, nutritionists and endocrinologists on research relating to women athlete health in Aotearoa (8) and co-produced an array of educational materials for coaches, athletes and parents (see https://hpsnz.org.nz/home/whispa-healthy-women-in-sport-a-performance-advantage/). She has also written on the theoretical and methodological complexities of such transdisciplinary research and practice (10, 11, 18), and the importance of localized and cultural ways of knowing health and wellbeing in elite sport (19). Sheree (she/her) was born in South Africa, grew up in Botswana, completed her PhD in Australia, and now calls Bath (United Kingdom) home. Her transdisciplinary research contributes towards a feminist Sport and Exercise Medicine, with a focus on sports injury prevention. As a member of the United Kingdom Collaborating Centre on Injury and Illness Prevention in Sport (one of eleven International Olympic Committee research centres worldwide specialising in athlete health and injury prevention), Sheree is part of a global network of scholars, clinicians and policymakers working towards health and wellbeing for all athletes. Her current research is comprised of two key strands: 1) understanding the influence of gendered environments on sports injury (7), and 2) conceptualising gender inclusive sport (20). She takes a translational approach to this research, with the aim of providing innovative considerations that are useful in policy and practice. She has also written on the practical complexities of feminist and transdisciplinary approaches, and the importance of recognising structural barriers (21–24).

We were motivated to learn from and with others, and hoped sharing this knowledge might support others seeking to do feminist research in and with a range of sports organizations. Recognizing the politics of inclusion/exclusion in women's sport, and feminist research practices, we took care in our considerations of whom to invite. Initially, we invited a range of scholars who i) identify as ‘feminist’, and ii) are actively working across the disciplines, with the aim to bring together a diverse array of perspectives in terms of geography, disciplinary backgrounds, and gender and cultural identities. We invited some feminist sport scholars from within our existing networks, as well as those with whom we had not yet met or worked with but whose work we admired. While some were unable to contribute due to other commitments, we were overwhelmed with the enthusiasm for this dialogue. We acknowledge that some perspectives (i.e., geography, disciplinary) are not represented herein, but feel honoured to bring together the voices of a diverse group of feminist scholars working in sports studies from a range of disciplines, including sociology, physiology, physiotherapy, and nutrition, and working with an array of national and international sports organizations. We also acknowledge that the questions asked and the ways this paper are presented have been shaped by our own experiences, assumptions and curiosities. While we have played an active role in curating this dialogue with the overall aim of creating a conversational ‘round table’ tone, we have also worked to respect the distinctive voices (i.e., word choices, communication style) of the invited contributors. We recognize this dialogue as offering only partial knowledge, but hope it opens opportunities for further conversations and action with those working and living across a wider array of geographical, social and disciplinary positionings.

Our invited collaborators and co-authors (in alphabetical order) include Professor Simone Fullagar, an interdisciplinary sociologist at Griffith University, Australia. Professor Fullagar has published widely on gender equity in sport, mental health, active communities and feminist new materialism (25, 26). Working at NtombiSport, South Africa, Dr Nonhlanhla Mkumbuzi's expertise is in sports physiotherapy, and is internationally renowned for contextualising sports and exercise medicine research, practice, and policy and her work in supporting marginalised athlete populations (i.e., women and girls of colour, youth, athletes with disabilities and those from low- and middle-income countries) to make evidence-based, contextually relevant decisions on their welfare. Professor Sophia Nimphius is the Pro-Vice-Chancellor (Sport) at Edith Cowan University (ECU) and maintains applied roles as a strength and conditioning coach and sport scientist. She has been involved in elite sport for over 20 years in Australia and the United States, leading improvements in sport performance environments, advocating for improving the sporting system for women in sport and the quality of research in exercise and sport science (12, 13). Dr Madeleine Pape is an Australian Olympian turned sociologist of gender. She is currently a Senior Researcher at the University of Lausanne, Switzerland, and engaged by the International Olympic Committee to support the rollout of their Framework on Fairness, Inclusion and Non-Discrimination. Dr Stacy Sims is an American exercise physiologist who has directed research programs at Stanford in the United States and AUT University and the University of Waikato in Aotearoa New Zealand, focusing on female athlete health and performance and pushing the dogma to improve research on all women. Professor Travers is a Professor of Sociology at Simon Fraser University, Canada. A central focus of their research is on transgender children and youth, as well as the relationship between sport and social justice, with particular emphasis on the inclusion and exclusion of women, queer and trans people of all ages.

In coming together in this dialogue, the intent is to amplify the voices of those working (empirically and clinically) at the disciplinary intersections of gender, sport and health, and thus to learn about some of the current and future possibilities for transdisciplinary innovations and strategies for building (responsiveness to) cultural change. Importantly, the contributors bring different understandings, interpretations and applications of feminism to the fore. The diversity of reflections, ideas and visions of the future shared by the contributors highlight the situated knowledge production and the role of locality that is often overlooked in global discourses on ‘female athlete health’. The locations, lived experiences and positionalities of the scholars included in this dialogue are not representative of all feminist scholars working in this field, but offer different perspectives that when read together, hopefully contribute towards more expansive ways of thinking about gender, health and wellbeing in elite sport environments, and towards approaches that celebrate difference, with a willingness to sit with the tensions, and ‘stay with the trouble’ (27). The remainder of this paper offers a curated dialogue to reveal some of the key passions, frustrations, learnings and strategies of this diverse group of scholars and change makers, and we conclude with some final reflections and future directions for sport scholarship and practice towards more gender inclusive, supportive and safe sporting environments for all.

## A cross-disciplinary dialogue: ‘Female Athlete Health’ and beyond

### Holly Thorpe

*In recent years, we have seen significant growth in interest in the gender data gap, and what has most prominently been positioned as ‘female athlete health’ in the various disciplines related to sports studies. What do gendered approaches to athlete health and wellbeing mean to you?.*

### Madeleine Pape

This is a timely question. I have personally seen in recent months growing interest amongst international sports administrators in developing a research and policy agenda for the advancement of “female athlete health.” I worry that this will follow the path of women's health research in the United States (US), where policy energy and resources have privileged a narrow focus on “biological sex” as the supposed foundation of women's health research (28, 29). In the US case, this has been the result of decades of mobilizing by feminist scientists and advocacy groups who embrace an essentialist vision of binary sex differences and, indeed, actively contribute to institutionalizing this vision of sex into policy. This has occurred despite key indicators showing a decline in women's health in the US relative to similarly wealthy countries over recent years––a phenomenon that clearly shows that women's health cannot be reduced to innate, biological differences between “the sexes,” and that structural and social factors must be playing an important role (30). We arguably face a similar challenge in sport: decades of investment by certain advocates of women's sport in a binary, essentialist understanding of female/male bodies, which was leveraged to create a “protected” category for women in what remains a male dominated and masculine world (31, 32), is now being translated into ideas about “female athlete health” that risk obscuring the role of the deeply gendered structures and practices of sport in shaping the health and wellbeing of all athletes. To me, gendered approaches to athlete health and wellbeing mean pushing back against binary, essentialist narratives and taking seriously the need to better understand how gender ideology in sport gets “under the skin” (33).

### Sophia Nimphius

To me and hopefully others, a gendered approach, particularly when discussing ‘female athlete health’, acknowledges the multiple influencing factors that have established the current understanding or lack of knowledge of the topic. I see the gendered approach as embracing the complexity, interactions and influences of often overlooked social factors and how they interact to exacerbate or protect one from biological attributes. A gendered approach to athlete health and wellbeing is to consider the interplay instead of the one-way biological “destiny” assumed and to recognise the immense influence that gender or one's gender identity, in addition to one's biology, has on athlete performance, health and wellbeing. I believe a gendered approach removes the hierarchy that biology is somehow exclusive in its ability to influence athlete health and wellbeing and that gendered influences are inconsequential. To take a gendered approach is to be holistic in understanding, examining and supporting our athletes. To recognise people are not robots with some pre-programmed biology independent of the impact and influence of their daily environment and surrounding social effects and expectations. Instead, people are immensely adaptable but also able to be influenced, and that must be considered within the social context to come close to advancing athlete health and wellbeing.

### Stacy Sims

As a physiologist it has been refreshing to finally have others in the field begin to understand that the female athlete is not just a smaller version of a male athlete; there are unique sex differences from birth, and again additional changes which occur at puberty with the exposure to oestrogen and progesterone. With regards to athlete health and wellbeing, it begins with research methodologies (scientific design through the female lens to include the hormone profile of the women being studied) and data interpretation- to reduce male biases and disseminate results in a manner that looks to improve female athlete potential. What I mean by this is currently the basics of coaching/training/recovery/nutrition are disseminated through research designed through the male lens, yet no one really questions if these basic fundamentals will serve the female athlete to achieve her best potential. For example, in this July's Tour de France Femmes (this was the FIRST Tour to be equivalent to the men's Tour de France that has been running since 1903!), was the first time professional female cyclists were under the same pressures with regards to difficulty of race stages, media coverage, spectators, etc. that the men have been under for decades. We saw performances from these women that no one ever could have imagined; another 3%–5% above what would have been expected (based on other major women's tours and single day races). Retrospectively examining the race, discussing with riders, and some of their directors, the undercurrent was that they all believed the potential for displaying what they were capable of doing had not ever been done but with this race, there was almost equality of media coverage (tv air-time, live stages being streamed, popular cycling sites covering the race as if it was a men's race). With this heightened exposure, the women were able to demonstrate that yes, it is a hard race, but when given the female environment, with coaches, allied health professionals, and support staff talking to the women as women, not as men, caring for their menstrual cycle health, mental health, and having open female conversations, there is so much more to be achieved in the sports space if (and when) the lens changes from male biases applied to female sport, to actually embracing the female environment. To me, when the environment around the female athlete talks to her and treats her like a woman (not a small man), the conversations open up around menstrual cycle health, mental health, physical health and adaptations which all lend to a holistic, healthy approach to superseding expected potential.

### Travers

Current approaches are problematic, as typical research and the typical model of gendered understanding, regarding sport is based on a two sex (binary) system, which, as we know, is ideological, rather than natural. We have a long history of research on male bodies only, and that doesn't necessarily apply to all bodies. So getting specific research on female bodies has, in some ways been effective but the full spectrum of sex identities isn't represented. This kind of research betrays a profound anxiety about clearly demarcating male bodies and female bodies, and that is at the heart of colonial heteropatriarchy. In this way, the real dilemma is how do you change the terms of understanding sex difference away from heteropatriarchal, binary sex systems, and have it be relevant and useful to those who the science going to benefit? We must also ask who the science is going to harm? And I think if we asked those questions instead of focusing simply on different sexed bodies we could engage in science that is more relevant and liberating. In general we need to think very critically about the science we are engaging in: How often does it have unintended harms?.

### Simone Fullagar

As a sociologist, I believe it is important to consider the nuance in how ‘gender’ is used in our thinking about athlete health and wellbeing across everyday practice contexts, policy frameworks and research approaches. This is where feminist and sociological insights can help to highlight i) how all gender categories (women, female, male, nonbinary, intersex, transgender and so on) are produced within an historical and cultural context, and ii) how they are embodied and lived (equity, health and wellbeing) within the power relations of a patriarchal society. In this sense a ‘gendered approach’ acknowledges the historical, sociocultural and biological forces that shape what athletic bodies ‘can do’ and how they are treated within the various systems and power relations that shape sport and society. Underpinning exclusionary power relations is a sex-gender binary (male/female) that has produced benefits for certain genders (white, cisgender, heterosexual, able bodied men have enjoyed significant privilege in sport), while cisgender women have had to fight for access, funding and representation because of systemic gender inequity. Such inequity is also exacerbated for marginalised women and hence a gendered approach also requires an intersectional lens that addresses race, sexuality, age, disability and gender identities beyond the binary (transgender, nonbinary and gender fluidity).

A gendered approach understands that oppressive power relations (overt and covert) in sport is the focus of change to enable different genders to become more than they have been told they can be. Embodied capacities can flourish through sport contexts that embrace equity and diversity. We continue to see gendered assumptions challenged in sporting feats and spectator growth – from Jasmin Paris' incredible experience of winning ultramarathon races in which men competed while she was breast feeding, to the huge fan attendance at women's Euro football matches in 2022. However, change is uneven for women of colour, with disAbilities, who are older and who identify as queer or transgender. Standing against oppressive intersectional power relations requires a feminist ethos that challenges practices of exclusion arising from patriarchal thinking that is damaging to women, gender diverse people and also men who are entangled in unhealthy masculinities.

### Nonhlanhla Mkumbuzi

A gendered approach is one that reflects that the sporting environment operates within a hegemonic masculine norm, with the adult man's experience as the default. However, we cannot ignore that different genders experience the sporting environment differently (7) and that we ought to accommodate these differences in the description of clinical features, development of training plans and clinical interventions. A gendered approach goes beyond the physiological and looks beyond hormonal fluctuations as the sole cause of sex related differences in injury risk, athletic performance, and overall experience of the sporting value chain. We cannot divorce the biology of sport from its socio-cultural contexts; contexts in which women and girls are treated differently because they are women and girls. With that in mind, we should acknowledge that while, for example, women and girls experience higher incidences of injuries such as concussion or anterior cruciate ligament ruptures, they do so in an environment that makes it more likely that they will get injured, not because they are inherently injury prone, but because of the way they experience the sporting environment (34). Women and girls often have poorer playing/training conditions, insufficient/ill-fitting equipment, dual careers, longer travel times to and from training/match venues, lower pay and lack of medical personnel, among others. A gendered approach to athlete health and wellbeing acknowledges all these in their management and that it is the way one is treated for being a particular gender, not the gender itself that is a risk factor for injury.

### Sheree

*Thank you so much for sharing your thoughts on this topic. Although you are coming from different perspectives, you are each so clearly knowledgeable and passionate about these issues. I am wondering if you could each tell us about the topic/s of ‘female athlete health’ or gendered approaches to athlete health and wellbeing that you are most interested in, and how you are working across disciplines to advance knowledge/practice in this area?.*

### Sophia Nimphius

As a strength and conditioning coach and sport scientist, I am most interested in the reduction of injury risk within the scope of ‘female athlete health’ or what I like to say is ‘female athlete performance’ because anyone seeking performance must inherently be assisting the athlete remaining healthy as the bedrock of performance. I suppose this has driven me to take a holistic or multiple discipline approach to female athlete health and wellbeing, the idea that performance is born of a foundation of health and wellbeing of the whole person. In my lived experience as an athlete and a practitioner, the concept of needing all aspects of yourself in harmony to maximise performance was innate. Still, as a researcher, I realised that we were quite myopic in our approach to explaining the causes of injury among female athletes (12). The narrative was a nearly exclusive focus on biological underpinnings related to the “female” aspect of the ‘female athlete’. As a result, I started questioning such approach to research design and research conclusions by ‘flipping the script’ and discussing aspects of athlete health (or performance) that were more readily accepted, such as:

*“if an athlete lacks the training age or history to develop sufficient strength, is this a key factor for injury risk?” The answer is almost unequivocally ‘yes’ from researchers and practitioners. I then ask, “are female athletes capable of adapting to training in a positive way that improves their strength and technical proficiency?” Again, the answer is almost unequivocally ‘yes’*.

At this point, I then question why the research design and conclusions immediately divert to our female athletes’ inherent inability as the cause for their injury rate or risk. Instead, a majority of research (and findings) gloss over or fail to discuss the training age, strength or a multitude of other training (and resource provision) factors that affect the development of any athlete. However, due to our gendered expectations (or lack thereof) of female athletes, we have set them up to be assumed to be the reason for their injury risk status, and we rarely dare to question the current system that “develops” these athletes. In considering all of this, it has been necessary for me to draw on my multiple discipline expertise to advance research/practice in the area and reach out to additional experts in psychology, physiology, epidemiology, sports medicine, motor control and sociology.

### Stacy Sims

As an exercise physiologist, my research and education is focused on a long list of topics, including Low Energy Availability (LEA), Relatively Energy Deficiency in Sport (RED-S), sex differences in concussion, Anterior Cruciate Ligament (ACL) rehabilitation based on menstrual cycle phases. Menstrual cycle health, Hormonal contraceptive use and effects on training and performance, Sleep changes across the menstrual cycle and into peri/post menopause; nutritional needs for the female athlete. I am working with femtech applications and platforms to educate and empower women across the lifespan on menstrual cycle health and nutritional guidance. I work with a major sleep tracking wearable and colleagues at UNC, Florida State University, and Stanford to dig into their large data set- not only to look at the data, but look to see where the algorithms are not capturing female physiology (specifically comparing menstrual cycle phases as there is a physiological change in the autonomic nervous system after ovulation; and again changes with OC use). In the deeper research context, I work with psychologists, data scientists, sociologists, physiotherapists, endocrinologists/medical experts; as well as epidemiologists. It is rare to work with another physiologist- I have been this way my entire academic career- seeking out the expertise in areas that complement what I am investigating, but where I do not have full insight or expertise. There is wealth and depth of insight that comes from understanding other disciplines' methodologies and views towards research/dissemination.

### Nonhlanhla Mkumbuzi

My areas of interest can be split into lab based and field based. In the lab, I'm working on a multivariate model to explore the influence of the menstrual cycle hormonal fluctuations on ACL injury risk in physically active women and girls. Here, we measure a number of variables such as hormonal concentrations, physical and psychological injury risk indices, lower limb mechanical properties and gene expression. This requires expertise from physiotherapists, exercise scientists, geneticists and psychologists. Additionally, I am interested in how breasts move during different exercise or sporting endeavours. This is a collaborative effort with sports scientists, biomechanists, biomedical, and electrical engineers. Lastly, I conduct field work together with epidemiologists and social scientists to explore the intersectionality of gender, race, culture, economics and environment in sporting participation, performance and rehabilitation. This is to better understand how the lived contexts of marginalised athletes, such as women and girls, interact with their biology and how this can better inform our clinical management and development of sport policy. Working with colleagues from these various disciplines allows a more holistic, multidimensional understanding of issues pertaining to women athletes that would not otherwise be obtained through a single discipline.

### Sheree Bekker

*Thanks so much Sophia, Stacy, and Nonhlanhla - you are clearly working at the cutting edge of feminist sport science, and the outreach work you are doing is very impressive. I might turn now to the feminist sociologists in our group to hear about their approaches to the topic of gender, health and sport. Madeleine, might you tell us a bit more about your fascinating research?.*

### Madeleine Pape

My research outside of sport has sought to better understand how “sex” as a so-called “biological variable” is co-produced through the political and scientific efforts of women's health advocates and biomedical scientists (29, 35). Key to this has been contributing to the efforts of feminist, queer, and trans scholars to reveal the complexity of what is commonly referred to as “sex,” which is not in fact an ontological “thing” that resides in the body. Rather, it can be understood as a system of classification that is applied to a collection of traits and processes that don't ultimately conform to a neat binary, nor behave in predictable ways: cisgender women's bodies are not one way, and men's another, but rather there is considerable overlap, dynamism, and indeterminacy in how the embodied traits we associate with “sex” present in bodies and come to matter to health and illness outcomes (36) (37). Some researchers might mistake for “sex” what is in fact be the result of social context shaping the body, which always already includes “gender.” There is exciting work that starts to chart the many pathways *via* which the practices and structures of gender can shape health, but we have much to learn in this area (38, 39). Here I believe feminist researchers studying athlete health can make invaluable contributions.

### Simone Fullagar

The gendered approach that I take in my research seeks to move beyond binary thinking that has dominated western knowledge in terms of mind/body, self/world, culture/nature, society/biology, masculine/feminine normativity. Our bodies are intelligent in all kinds of ways when we learn to listen, just as our minds and emotions are entangled with visceral qualities of moving embodiment. Through sport our hearts race with anticipation, knots of fear are felt in our stomachs and the pleasure of moving with skill or flow can transform our thinking-feeling states. This bodymind movement is also profoundly entangled with the gendered contexts of our sporting lives – the memories that haunt us, relationships that support or harm, the shame and pride we learn as children in the face of expectations to win, enjoy or be recognised by others as normal, good, desirable…

In relation to sport and embodied movement I am particularly interested in rethinking assumptions that have shaped mental ill health, such as gender neutrality and individual mind/brain disfunction. From a feminist perspective on embodiment I explore how experiences of distress and recovery involve bodyminds, affective relations of power (thinking-feeling about oneself in a gendered world) and contextual relations of inequity and support (people, places, digital technology etc). Our bodies exist as an individual-social, cultural-biological nexus where gendered power relations play out in ways that are felt through distress and trauma. Yet such experiences are often individualised through shame and self-blame relations (what is wrong with me/them?), rather than recognised as a response to inequitable sociocultural conditions (how has this distress materialised through this gendered context?).

Within contemporary mental health promotion, sport is expected to be a site of positive wellbeing yet the scenario is far more complex when we look at how distress can be shaped by sport contexts (such as, high performance cultures, body perfection, abuse, racism, ableism, sexism, homo/transphobia and so on). A transdisciplinary feminist approach to mental health acknowledges that psychological perspectives benefit from the contextual insights of sociology, which in turn requires understanding of the somatic and epigenetic dimensions of embodied distress and trauma. Without a gendered approach to sport and mental health the fields of research, policy and practice will continue to overlook the crucial relations of power that can erode wellbeing, or enhance capacities for collective and individual support.

### Travers

My work addresses the white supremacist foundation of gender categories. When I published a piece in the *Journal of Sport and Social Issues* in 2011, about women's ski jumping and the 2010 Olympic Games, I asked sport scholar Delia Douglas, a friend and colleague, to read the paper. The paper at that point chronicled the court case in Canada about the women being excluded from the Olympic Games. The draft included a minor commentary on the White supremacist function of the winter Olympics as a celebration of Whiteness and wealth, however the emphasis of the paper was on sex differentiation. Douglas' intervention was to challenge me to really unpack and dig into the former - the White supremacist foundation of the winter Olympics, and it is one of the most valuable interventions anyone has ever made in my scholarship. I'm deeply grateful to her for that. It has shaped and pushed me in ways I needed to be pushed, and it was very generous of her to do so. It must be so exasperating and exhausting for scholars of colour to run up against the myopia of their White colleagues all the time. So her generosity in making this intervention in my scholarship was deeply appreciated. I was also incredibly influenced by Sherene Razack, who is a Canadian scholar who teaches us that gender has racial logics, which I understood, but Delia Douglas helped me put it together. Douglas and Razack helped me develop a much deeper understanding of the ways in which gender and race are co-constituted in the logic and materiality of colonialism and narratives of European ‘civilization.’. And then I wrote about baseball subsequently, using the frailty myth (40) as a white supremacist heteropatriarchal notion. When I wrote my book on trans kids in 2018, I used assemblage theory emerging from Black feminism, reading Sylvia Wynter, Alexander Weheliye, Hortense Spillers, Audre Lorde, and Patricia Hill Collins (arguments related to overarching Eurocentric biopolitics for which Foucault often gets undue credit). As a basis for my own intellectual development, I started a critical race, feminist technoscience reading group, and we started out with an excerpt from Habeas Victus by Alexander Weheliye. And then we read Sylvia Wynter. This led to my development of a graduate course a few years ago, called Whose Lives Matter? In this course, we exclusively read social theory by scholars of colour/Indigenous scholars. So that was how I moved into writing about transgender kids in the context of racial capitalism and colonial narratives of civilization, understanding gender systems in this context. This work built on the scholarship around sex segregation and sport that I started to engage in in 2006. I was deeply curious - and disturbed by - potential overlaps between racial segregation and sex segregation in sport. However, it was not until I started to deeply understand the co-constitution of White supremacy and heteropatriarchy, in the context of colonialism, the trans-Atlantic slave trade, and Eurocentric civilizing narratives, that I was able to contribute something of real value. I feel that my scholarship has really matured in the past decade or so.

Over the past decade It has really come together for me, how white scholars are inoculated against our own privilege; Patricia Williams (41) definition of power as the right to not know is a real touchstone for me. I have become more aware of White wilful ignorance and started to really unpack it, to confront the notion of civilization - the ideological justification for colonialism and genocide - which is foundationally white supremacist and hetero patriarchal. In Black feminism, woman of colour feminism, anti-/postcolonial and anti-racist scholarship in general, these conversations have a long history, categories and relations of oppression are assembled together, which is the contribution that assemblage theory makes. My insights in this regard are far from original. But my application of assemblage theory to issues related to sex segregation and transgender participation in sport is groundbreaking. Applying assemblage theory, *via* theorizing relating to theorizing regarding necropolitics (42) and queer and trans necropolitics (43, 44), in particular, to Sport Studies, is very exciting for me. Understanding the emergence of modern sport and its contemporary articulations within and as part of this assemblage of oppression is driving my current scholarship on sport.

### Holly Thorpe

*Wow, I am so inspired by the incredible work each of you are doing, and the different ways you are working at the intersections of gender, health, science, race and ethnicity. What are some of the different disciplines you have worked with in your inter-cross-transdisciplinary work? Could you tell us about the logistics of working across these disciplines? (i.e., how did the collaboration come about; how do you work together on this topic of shared interest).*

### Nonhlanhla Mkumbuzi

I trained as a physiotherapist, then exercise physiologist and exercise scientist after that. Across these disciplines, I have gained expertise in clinical physiotherapy, small animal surgery, microscopy, DNA profiling techniques, and ultrasonography, among others. Consequently, my research processes have always tended to be multidisciplinary and to be informed by these various fields, which leverages understanding of sporting phenomena from a molecular level to the clinic. Hence, the leap to collaborate with colleagues from other disciplines such as cell biology, through social sciences to electrical engineering was a natural progression that enable me to participate in research that explores the totality of the human experience in all its complexity and ‘messiness’. The human experience is not unidimensional and viewed from different lenses always has something new to show. Therefore, we do not do the study of women athletes justice by studying them one discipline at a time as this limits our understanding of them. My collaborative experiences have been nothing short of incredible. Not unchallenging though, conversations with the electrical engineers are essentially in a different language, and the qualitative methods of the social sciences sometimes go over my head, but we make it work and we hope to understand women athletes much better than we would if only sports scientists were studying them.

### Sophia Nimphius

I have benefited from the knowledge and expertise of people from an enormous range of disciplines, from biomechanics, data governance and statistics to sociology and psychology to physiology and motor control through to sports medicine and epidemiology, and I'm sure many more! To say combining all of these areas into a single piece of work or research is easy would be false. Still, research isn’t easy because life isn't easy, so it is something I have accepted that just takes time but is what I think will ultimately make the most significant impact. Simply stated, we can and should do hard things! The logistics of doing so are admittedly complex. However, I am extraordinarily fortunate to have had a life and educational path of learning and thinking from different disciplinary lenses. I state lived and educational because one cannot separate the person from the research. You borrow from what you've experienced and that you've been formally taught, in complement, not a contradiction of each other. My lived experience of the influence of society was initiated by navigating life as an openly queer, Latinx woman in a male-dominated sports industry and even more so strength and conditioning and as an athlete in the US (including the NCAA system) and Australia. I have formal degrees in sport management, biology, strength and conditioning research and sports science. The result is that I've “learned the language” and “earned the trust” of many different disciplinary lenses enabling me to understand the needs, expectations and biases of each discipline and be a link between fields when performing inter-cross-transdisciplinary work. Therefore, almost every one of the projects I have been able to lead or organise is of this merged approach to understanding. This perspective of trying on multiple lenses is one I try to pass on to my post-graduate students that, much to their editing displeasure, we often have supervisors from a range of discipline expertise. For example, in one project, we have a data scientist, biomechanist, motor control and skill acquisition expert, psychologist, strength and conditioning coach and me as the ‘linking communicator’ as we seek to re-evaluate movement assessment. The approach came about because I recognise each discipline's value in contributing to the big picture of our problem, and I respect those perspectives. In short, people are always happy to contribute when they are clear from the start that we are coming to the project to contribute multiple perspectives, not to form an echo chamber.

### Stacy Sims

The collaborations across the different projects came from exploratory interests and the common thread of furthering sound research on female athletes. Most of my colleagues on these projects are international- thus we have regular virtual meetings, and, when possible, in person meetings (Which is more available now that the borders are opening!). We usually divide different sections of the research according to our expertise, then come together to disseminate and educate the other members of the team of how/what we have done/found. It is a fantastic way to learn when we are already at a point in our careers where others are looking to us for the methods and answers. I will say that my favourite cross-disciplinary/transdisciplinary projects are with sociologists and psychologists- so much to learn from them and change the way I think about research design and methodologies. I do not think I could go back to straight exercise physiology, surrounded by exercise physiologists- it is too one-dimensional and misses the nuances within research that can result in novel outcomes and dissemination.

### Simone Fullagar

I started my early research collaborations on women's mental health with an incredibly generous feminist psychologist (Suzy Gattuso) who was a great mentor and colleague. I was working on a project related to the social context of youth suicide and she had just finished an oral history project on women's narratives of ageing. We both identified the gendered conditions of women's depression as a research problem that needed further investigation in order to change popular and professional understanding. We began this across a range of projects related to media representation (women's magazines), individual and cultural narratives (qualitative projects), community intervention evaluations (women's health project using leisure and fitness) and so on (until Suzy retired). I have also collaborated with creative arts practitioners to research how dance and movement are experienced as gendered practices through different positionalities (queering knowledge). These collaborations worked to bring our very different theory-method approaches into new feminist configurations and spheres of influence (dance, fitness, leisure, sport). Collaborations ‘do things’ to our ways of thinking, feeling and acting that are surprising, at times frustrating, but most often are rewarding in terms of the synergies produced.

### Madeleine Pape

Though the work is still relatively nascent, some of the most exciting work I've been involved in has been a collaboration across sociology, sports medicine, law, gender studies, and leisure studies to map out a vision for sports organizations of “gender inclusive sport:” sport that is inclusive and affirming of all sports stakeholders, and which challenges the cisgender, essentialist binary organization of sport in ways that delivers equality for all––including for cisgender women (20). Thanks to this project, I have seen that I have work to do to broaden my own sociological research agenda to ensure that I am not only diagnosing the problem but that I am also coming up with practical solutions. I think it is in this move––towards practical solutions and charting out real pathways forward––that inter-cross-transdisciplinary work is key.

### Sheree Bekker

*What a remarkable array of inter- and transdisciplinary projects you are involved in. What do you see as the strengths of working across the disciplines?.*

### Stacy Sims

From my experiences, I would say that the absolute strength of working across the disciplines is in the different visions, interpretations, and ways of conducting research- from recruitment language and strategies, the data collection environment, to data analysis. Again, working across disciplines allows a depth of research to emerge that otherwise would be missed or lost, as well as designing studies to dig into areas that might also be missed or put aside as outliers.

### Nonhlanhla Mkumbuzi

Working across disciplines allows us to gain greater understanding of any phenomenon. For example, my work on the intersectionality of biology, race, gender, culture, environment, and economics can only be done through multidisciplinary collaboration. Put simply, there is no one discipline that would do the topic justice. Additionally, a multidisciplinary team benefits from different methodologies and paradigms, each with their own strengths and each allowing us to understand various aspects of the same issue. While a geneticist is well equipped to answer how gene expression in the genes that control muscle metabolism changes across the menstrual cycle, they are ill-equipped to explore how muscle strength changes throughout the month, or how societal attitudes to menstruation impact sporting participation in women and girls. The only way we can understand how all of these interact in a woman athlete, to ultimately influence injury risk or athletic performance, is by engaging the expertise of a multidisciplinary team to each explore the subject from different viewpoints using different methods.

### Sophia Nimphius

For me, the strength of working across disciplines is using different perspectives to shine a light on a unique or potentially interacting series of factors that may be a vital contributor to the problem or question that is being asked. When a group of different disciplines are in the room, and the contributors are open to listening, I find you learn from their perspective. You become more creative and less defensive, and it aligns you with the true intent of the research, which is to contribute to the knowledge and understanding of the topic, not to be personally responsible for being the “correct” person in the room. In short, while this approach can slightly increase conflict (in a positive way), it dramatically improves creativity and problem-solving to have this diversity of thought. Further, I believe the benefit is ensuring the conclusions are moderated so that the magnitude of effect is proportionate to the claims that are made. This becomes particularly important when discussing ‘female athlete health’ where there is vast variability of individual physiology and social influence depending on the cohort investigated. As such, our discussion, magnitude of impact and generalisability of research conclusions in context will only benefit from research in this area working across disciplines. However, I have yet to be able to fully transition from this multi- cross and interdisciplinary to truly transdisciplinary. I suppose that's the next 20 years!

### Madeleine Pape

In my work on the regulation of eligibility in women's sport, I see how harm to athletes has been sidelined as a “secondary” concern vis-à-vis the scientific question of what constitutes “sex” and disproportionate advantage in sports performance (23, 24, 45). I currently work at the IOC to support the rollout of the 2021 Framework on Fairness, Inclusion and Non-Discrimination (46), where it is an ongoing project to ensure that the health, wellbeing, and bodily integrity of athletes potentially impacted by eligibility criteria is recognized as a consideration that is central rather than peripheral to the debate. Currently there is a vast inter-cross-disciplinary network of researchers and advocates working together in an informal way to compel International Federations (IFs) and the IOC to make good on their commitment to inclusion and non-discrimination. There is no question that these inter-cross-transdisciplinary efforts have had impacts. However, I have recently been thinking about how we might scale up our collective impact by better organizing our work, such as *via* a formal alliance. The forces invested in the binary, essentialist, heteropatriarchal organization of sport are strong, and the system is set up to disadvantage those of us who wish to change it. In my view this requires new structures, which should not only be inter-cross-transdisciplinary in an academic sense, but also bridge academia and practice.

### Holly Thorpe

*So, you've made a very convincing case for the benefits in working across disciplines to advance new ways of understanding ‘female athlete health’ and gendered experiences of sport. Of course, this sort of work can be very difficult too. Might you share with us some of the challenges you have experienced in working across the disciplines, and could you possibly share any strategies you have developed for doing so?.*

### Simone Fullagar

A strategy that I've found helpful when working with scholars and practitioners from different disciplines is to start by articulating how feminism is not just about empowering women, it is about producing different ways of knowing the world and understanding what gender assumptions ‘do’, in order to intervene. How do we start thinking this challenge together and also acknowledge our blind spots, privilege, disciplinary blinkers and importantly points of connection across ideas, methods and translation practices? A gendered approach can provide the language to think beyond siloed disciplinary perspectives that assume gender is either a social variable or biological sex (that somehow exists outside the culture used to understand it). I am always conscious of not reinforcing powerful biologically based discourses about ‘female athletes’ (fragility, cisgender, heteronormative) so I prefer to use the term gendered embodiment rather than sex/female categories. Gendered embodiment also acknowledges how sexed bodies are composed of biology and culture in historical ways that are more complex than is often acknowledged.

### Sophia Nimphuis

The most overwhelming challenge is that there will be more conflicts when working across disciplines. The key to this challenge is becoming comfortable in uncomfortable, which can be immensely difficult for many, depending on their prior experience in being questioned or challenged or not having power or priority in a discussion. I probably have a lot of strategies depending on the situation. I'd say that there are many people with similar expertise, so choose the team for their expertise, trust and interpersonal skills. To be blunt, there is a ‘no asshole policy’. Like any team or relationship, some are transient so re-evaluate multiple discipline teams as you progress over the years. Second, be open and honest with expectations. If you are the key organiser of the group, then lead by example by showing humility, giving recognition and being constantly curious about each team member's expertise. As a pause to this, when genuinely dedicated to advancing an area and improving diversity, equity and inclusion, be prepared to learn continuously and humbly take feedback on inclusive language. If you can't, you probably aren't in this for the right reasons. Finally, after what seems like me saying, “*can’t we all just g*et al.*ong”*, realise we must eventually take a stance and, in that stance, be respectful and acknowledge that the decision, outcome or explanation the team decides upon will likely fall short of any one person's “preferred narrative” but that this is just where our research lands us at that time. In a few years, decades or more, we expect that the research approach, conclusion or explanation on the topic will and should evolve from what we decided on, so don't put too much pressure on each other or ourselves. Our job is to move the research forward, not for the work or paper to be the final guide on the topic ever to be written. Science is progressive, eventually. In taking this last strategy, we become less attached to ‘how I would’ve said it’ and more focused on ‘moving the needle forward for where we are now’.

### Stacy Sims

The biggest challenge has not been across disciplines, *per se*, but androcentrism. My male colleagues have been/are great for the most part, but so many of their entrenched methods of conducting research are through the male view that has long dogged biomedical research. Several sticking points have been in recruitment/recruitment language- and by using examples of how women may feel when confronted by certain words or attitudes, we have been able to change the recruitment messages thereby increasing interest, recruitment, and attrition to studies. Other challenges have been in the lab itself- with human performance research, there are many uncomfortable things we ask participants to do- nude body weight, blood sampling, urine sampling- and many of our projects centre around the menstrual cycle, thus collecting samples during the menses phase. Reducing emotion of the research assistant around the specimen (eg. Ooh.. there is blood in the urine…) and applying a female view to what is being asked has reduced the discomfort of both participants and research assistants. Other challenges have been walking into a total male environment (gym, orthopaedic surgeries) as the only woman, and being dismissed as the lead research scientist- still trying to navigate that one.

### Travers

My biggest challenge is my own short sightedness. You know how it is: you don't know what you don't see until somebody points it out to you. I was never comparing them exactly, but when I started talking about sex segregation in sport having something in common with racial segregation, I started presenting my work on this topic at the North American Society for the Sociology of Sport Conference, and all I got was pushback. I got pushback in particular from queer white feminists. And whilst I do think some of it might have been because of the immaturity of my scholarship at that point - I had SO much to learn - I was not suggesting that we equate racism and sexism, but rather: Why is sex segregation okay when racial segregation isn't? Both are based on socially constructed categories of difference as the basis for oppression. This was my own initial attempt to reckon with what meaningful intersectional analysis looks like. So I started doing these Challenges to the Gender Binary in Sport sessions and there was such strong interest. I remember one session, I think it was in 2004 in Tucson, that was scheduled for 8am on the last day of the conference and it was packed. I ran these sessions for years and attendance was always high: there was obviously a real thirst for this. But in terms of getting published in the Sociology of Sport Journal, I couldn't get published. As a result, at that time, I definitely felt like, yeah, I was not cool. I was not in the in group of queer feminist sports scholars at all. So you can only imagine how good it felt years later when a colleague sent me an email stating “Travers, you were ahead of the curve on this one.” And later a colleague of mine said: “you were right all along. Now everyone is talking about what you were one of the first to talk about.” My coping strategy at the time was to go outside Sports Studies and I published my argument about the relationship between sex segregation in sport and gender injustice in the *Studies of Social Justice Journal*, in 2008. I'd come across an article in this journal that I knew I could use as a jumping off point, written by Nancy Fraser in 2005. Fraser defines gender justice in terms of both recognition and material resources; gender justice therefore requires both cultural and material redistribution. So I was able to talk about sports in this way, and it just worked. If you're doing good work, you're going to get it published somewhere. I don't think it's an accident that it was harder for me to get going, but also learning to write for publication is a craft. I am a much stronger scholar now than I was when I was beginning to investigate these topics.

In tandem with the sessions I ran on Challenging the Gender Binary in Sport at NASSS, I co-organized sessions called Race and Gender in Sport: Intersections, with African Canadian scholar, Robert Pitter. Robert was intrigued by my work investigating similarities and differences between forms of segregation in sport and really encouraged me. We connected about the lack of attention to gender in sessions on race and sport at NASSS and vowed to try to bring mostly male scholars of colour into conversation with queer and feminist analyses. With the notable exception of Delia Douglas' brilliant work on Venus and Serena Williams, most of the scholarship concerning race and sport failed to attend to issues of gender. It was at NASSS that I met Delia when she presented her work on the racism directed at the Williams sisters at the Indian Wells tournament (2004, Sociology of Sport Journal). I also connected with African Canadian scholar, Robert Pitter, who became a dear friend and co-conspirator as we worked at NASSS to bring conversations about the intersections between gender and race together.

### Nonhlanhla Mkumbuzi

The biggest challenge working across disciplines, be it in clinical practice or in research, has for me been ‘prioritisation’, if I can call it that. Which discipline do you ‘prioritise’ at any given time. As a multidisciplinary clinical team, we each come from different perspectives and have seemingly different objectives to achieve for the team or athlete. Sometimes those objectives may be at odds with each other, which may lead to conflict over which one should supersede the other. For example, the coach might want their star player to be in the first team line up, while my clinical opinion is to rest her. How to have that discussion with both the player and her coach has often been a challenge for me as I try to balance the coaches' objectives to win and the clinical team's one to promote player welfare and wellbeing in an environment that has other dynamics such as power differentials and vested commercial or national interests involved. Similarly, in a research team, balancing a multidisciplinary team's various paradigms and narratives in a research study or manuscript publication can be a challenge. For example, the menstrual cycle and menstrual hygiene management can be explored or discussed from a purely medical, sociological, or economic narrative. But in an instance where all those disciplines are involved in one project, how do we balance each discipline's methodologies and narrative to ensure intersectionality is well represented? In both the clinical or research cases, I agree with Sophia that we need to respectfully embrace the discomfort that will inevitably come with these differences. We also need to have pre-determined conflict resolution plans. Having these decided upon before the heat of a conflict allows us to have something structured to fall back on should we need it. We also need to tailor our messaging, clinical or research, to our audience. This helps us determine which narrative to ‘prioritise’. Above all, we ought to always keep our ultimate goal in mind, as clinicians on a sports team, our mandate is to get the best performance out of our players while maintaining their welfare. As researchers, we want to advance the research, and by extension management, of women and gender expansive individuals in sport.

### Madeleine Pape

On the topic of eligibility regulation, a key challenge is convincing the sports science and medicine community that they, too, should not just value but prioritize athlete health and wellbeing. When IFs are making decisions about eligibility criteria, they grant enormous power to sports doctors. What I find interesting is that in the context of sport, doctors have a kind of double hat: they have a clear responsibility to prioritize athlete health (47), yet they also value (and typically prioritize) the sports science pursuit of “performance” knowledge (48). The struggle, then, is to articulate claims about the harms of eligibility regulation that are legible to sports scientists and doctors. A key challenge in my current role at the IOC stems from the realization that being adversarial is not necessarily productive. The influence of sports doctors and scientists on how IFs approach eligibility criteria hasn't just disappeared overnight as a result of the IOC Framework. This is the reality. As a result, at this stage in my role it is necessary to try to work with sports scientists and doctors in trying to find where the common ground is and think strategically about what we can and can't achieve at this moment. We need to plan and prepare for the long game.

### Holly Thorpe

*What a brilliant collection of responses. The challenges you've identified vary from the androcentrism of sport science, to the push back from some groups of sport feminists. And you've each been very generous in sharing some of your strategies for working more effectively with colleagues from different disciplines – I love the ‘no asshole policy’ – to starting with some definitions about what feminism is and how it might productively shape your collaborations. Personally, I also really resonate with Madeline's observation that ‘being adversarial is not necessarily productive’, and I think this can be quite challenging for feminist sport sociologists (well, speaking for myself here) [**Sheree:** same!] who are committed to working towards cultural and systematic change, but need to find strategies to help move others along without evoking that ‘knee jerk reaction’ that often tends to close doors rather than encourage others to lean into processes of change. Personally, I am still working towards ways of productively converting rage/disappointment/frustration into constructive activism, so it is very heartening to hear of your different approaches. I am wondering now, what do you believe are some of the most pressing issues facing women and gender expansive people's health in sport at the moment?.*

### Travers

I think one of the things that we need to look at is the impact of transphobia on cisgender girls and women. Athletic and muscular girls and women have always faced ‘questions’ about their gender and sexuality so this is not new but high profile controversy around female eligibility policies in sport that have disproportionately targeted Black and brown women from the Global south and highly organized and well-funded campaigns, by an alliance of White, Christian ethno-facist groups and trans exclusive radical feminists, to prevent trans girls and women from participating in sport in the U.S. and the U.K. are contributing to a hostile climate for all girls and women.

At the same time that sport scholarship needs to integrate its understanding of the co-constitution of gender and race, I think the analysis of the relationship between sport and racial capitalism really has to be brought to bear. You can't understand the careers of Serena and Venus or Colin Kaepernick, Lebron James, or Michael Jordan, without understanding racial capitalism. The way in which racial capitalism is operating through sport, I would say, is one of the most significant areas for critical scholarship. So much about that needs to be understood. Like, what kind of inclusion is afforded in sport as it is currently constituted?.

Beyond being enraged by the ways in which sport reinforces brutal hierarchies, however, there is much about sport that excites me. I love baseball, I can't help myself, although my experience as a youth baseball coach reads like a primer in the operation of heteropatriarchal systems of power and their ability to adapt to and maintain themselves in changing social contexts. I am deeply gratified to see Serena Williams being widely celebrated, however inadequate the attention to the racism she has experienced. And I can't begin to contain my excitement about the way that the WNBA has been transformed by its players over the past decade to celebrate and include gender and sexual diversity, and to be both a forum for anti-racism and a locus of anti-racist organizing. The WNBA has shifted over the last decade from the usual homophobic ‘don't ask, don't tell’ to inclusion and celebration of queer and Black women. Fuck yeah!.

We've seen the impact of white supremacist and racist gender norms in terms of media coverage of Venus and Serena Williams, for example, although as she ‘evolves’ away from tennis Serena seems to be finally getting credit for being, truly, the greatest tennis player of all time (of any sex). But right now I think we're seeing a backlash to the ways in which the ideology of two sex system has been challenged by transgender people in general and trans women in particular, and women whose sexual markers defy simplistic categorization. The failure of ‘science’ to demarcate clear boundaries between only two sexes is an absolute, and too long in coming, crisis for Western, or, as Indigenous musician and activist, Buffy Ste. Marie, describes it, ‘White’ culture.

### Madeleine Pape

Building on Travers' response, I would identify my most pressing health issue in sport as sex testing, and particularly the sex testing of women of color from the Global South. I have personally sat across the table from an athlete whose life and sense of self was destroyed from the decision, shared publicly, to ban her from women's competition and void her results following a “failed” sex test. Sports organizations and their chosen experts like to claim on paper that an eligibility policy can be drafted in a way that avoids harm (read: avoids responsibility for harm). Appeals to informed consent are used to justify whatever actions follow, as though women in such a situation could ever freely consent to the medical examinations and procedures deemed necessary by their sport's authorities (49). The IOC finally acknowledges that there is no such thing as a test of “sex,” and that such practices result in irreparable physical, psychological, and social harms to the affected women (46, 50). This in and of itself is a victory. However, new cases of so-called sex testing continue to emerge with immeasurable consequences for the women impacted and, at the time of writing, the IF for the sport of swimming has released a new eligibility policy for women's competition that reintroduces mandatory sex testing across all women swimmers (51), a practice thought relegated to the dustbin of history by the mid-1990s (52). The persistence of sex testing as an institutionalized practice in sport puts many women and girls at risk of experiencing invasive gynecological exams and unnecessary medical interventions including irreversible surgeries, as well as the emotional and social harms that can stem from an unnecessary “diagnosis” of intersex variation (53–55, 45). This issue should be at the center of efforts to ensure the health and wellbeing of all women––and of all people, period––in sport.

### Simone Fullagar

The prevention of violence, harassment and disrespect in our sport systems at all levels is crucial. The related issue of mental health needs to be understood a gendered phenomenon, not as an individualised problem as that can do more harm than good. The spotlight needs to be on changing dominant masculinities that devalue and exclude women, gender diverse people and nonhegemonic men, as they are part of the power dynamic that undermines wellbeing and prevents alternative ways of becoming through sport and transforming it.

### Sophia Nimphius

I would have to say a key issue may be that we are starting to turn to sport to fund and discover solutions about even the general health of women without questioning how the health of women has not been prioritised by society as a whole and the funding structures of governments to support the health of women. Further still is how far behind these same systems (institutional and government) are in providing and understanding the health of gender expansive people. A key issue is finding the line, which will inevitably be blurry, where a proportionate amount of responsibility lies. It is a real problem that the sporting system and bodies that should support sport for women and gender expansive people were already behind in just providing equal sporting opportunities. The pressing issue is how they also attempt to research and understand health with the already disproportionate funding to women and gender expansive people in sport. Secondly is the currently proposed battle of ‘fairness’. The newfound enthusiasm to “protect” women in sport is diverting an extraordinary amount of attention from fundamental issues in sport of respect, equity, resourcing, athlete protection and a wealth of other areas. More significant progress would be made if a similar magnitude of media attention and resourcing were positively directed to these issues vs. scapegoating some of the most marginalised people in our society. So, there are numerous issues that we need to pay attention to, and in doing so must be careful not to scapegoat people to protect a system that may be using a distraction tactic to shield from greater scrutiny and uncovering of systemic failures. It is not us vs. them; we all deserve health and safety in sport.

### Stacy Sims

There is so much chatter around the menstrual cycle- and the “lack”of research to apply guidelines vs. “emerging” research to support guidelines with individual nuances. This is confusing so many athletes as to what the menstrual cycle means- and what does it mean to be “healthy” -eg not having a cycle vs. having one; both in the cis and trans environment. Before we can all move forward, more research needs to be conducted from the molecular level to whole body and mind, to identify what it does mean to be a *well* female athlete. As hormone influence is just one small part of the conversation, we need more transdisciplinary work to identify the social, psychological, and cultural aspects of what drives women to sport, and the methods of training to promote performance potential without androcentrism in the environments that surround the athletes.

### Nonhlanhla Mkumbuzi

I think in general, lack of investment is a huge challenge for the health of women and gender expansive individuals. While men's sports are heavily invested in, women's and girls' sports are woefully undercapitalised. As a result, health issues that pertain to women and gender diverse individuals are not researched or prioritised as a matter of course. At best, these are niche, at worst they are ignored altogether. Consequently, because of this lack of investment, we lack the resources, financial or otherwise, across the sporting value chain to enable us to provide the best support for women and gender diverse athletes. It seems women's sports have to prove themselves worthy of investment first before people are willing to invest their time, effort, and money at any level and in any domain. There is no better reflection of this than the upsurge in interest in women's football in England. Women have played football in their domestic league for decades now but match attendance by spectators, interest and coverage from the media and the resultant income generated and support for the players was very low. Now they are breaking attendance records at their matches and interest in the game has exploded. It seems all they had to do was win the UEFA Women's EUROs first.

### Sheree Bekker

*This work can, understandably, often feel and sound like bleak, futile work. What, in your experience, is needed to achieve more holistic and multidimensional health care for women and gender expansive person's in sport?.*

### Nonhlanhla Mkumbuzi

To provide holistic care, we need to always have the person at the centre of all our care. That means always considering their whole experience in our management. Our athletes are more than bones, muscles, and world records. They are also the sum total of their life experiences. Experiences that are shaped by their gender, race, culture, environment and socioeconomic status, among other things which cannot be separated from their biology as we care for them. In order for us to provide holistic management for women and gender expansive persons in sport, we need to always have an intersectional lens to our clinical management and policy development.

### Sophia Nimphius

We first need to continue changing the breadth of voices that drive the decisions at every level of sport, from community to elite to research. Unfortunately, despite the calls for this change, it is prolonged and will likely mirror the change timelines of similarly large institutions such as the government, which acknowledge that even for “gender equality” the wait is beyond 100 years. However, we often stop and measure our success just by a gender equality that has largely left gender expansive and intersectional voices out of the discussion. To achieve more holistic and multidimensional health care, we must extend the voices and power to a much greater cross-section of backgrounds of people to enable the diversity of thought required to tackle the enormous task of delivering health care to all our women and gender expansive persons in sport. In addition, we need to learn from past efforts to improve health care and any aspect of sport for women and gender expansive person's. For example, in the United States, when Title IX was passed, paving the way for the enormous rise of participation of women and girls in sport, this ‘professionalisation’ led to decreased head coach positions being occupied by women for these now higher remunerated and more prestigious jobs. Before 1972, more than 90% of collegiate athletic women's teams had a woman as head coach, dropping to less than 50% by the 1980 s and even more disturbingly remains at that level today (56). I plead that we learn from what can occur with the professionalisation of sport for women and girls and state that when changes occur, we must also consider the leadership that is put in place to help drive this change. The leaders need to be representative of the people they seek to make improvements for, so I suppose my thought is our greatest need is not to make the same mistake with leadership as we did with coaches after Title IX. In this next wave of improving the health of our women and gender diverse person's in sport, we must ensure the leadership has a history of working with the relevant population or is representative of the population they seek to lead.

### Madeleine Pape

To me, this work must start with a multidimensional theory of gender that recognizes that biology is inseparable from social context, and that an essentialist, binary, heteropatriarchal ideology of gender is built into our sports institutions in ways that are then expressed in and through all of our bodies (57). This will mean different health impacts for different people, depending on their relationship to this ideology as well as on where they are located in sport vis-à-vis this and other institutionalized axes of difference and inequality, perhaps most notably able-bodiedness and whiteness. This is where the “female athlete health” agenda risks starting off on the wrong foot: if it does not begin from a systemic account of gender, it may never account for the diversity of ways that gender shapes different athletes’ biosocial experiences. Does height rather than sex category explain rates of shoulder injury across differently gendered people (58)? Do poorer ground conditions rather than sex category explain differences in knee injury in professional women football players (7)? How do male-identified athletes experience harassment and harm in sport settings (59)? Rigid categories do not serve us well in seeking to ensure that sporting environments advance the health and wellbeing of all athletes.

### Simone Fullagar

We need more interdisciplinary feminist dialogue involving researchers, practitioners and policy makers to recognise how different kinds of expertise and embodied experiences can knot together into new framings of issues and ways to address change. Funding for these kinds of projects is crucial and involving sport organisations in the collaboration can produce different kinds of evidence that can be shared and translated into a range of practice contexts.

### Holly Thorpe

*Our final question now is a biggie: What steps do you believe are necessary to build more inclusive and supportive environments for women and gender expansive people in sport?.*

### Travers

Public funding. Part of neoliberal globalization has been the retrenchment of community based sport programs. We focus so much on elite sport participation, but that is not what really matters in terms of opportunity and enjoyment. Since 2008, I have argued that we need to eliminate all boy and male only sporting spaces and have an open category, and then have a girl and women only space that is trans inclusive. I've looked at lesbian softball leagues as a model for that, because I don't think that exposing girls and women and gender nonconforming people to misogyny is the answer to throwing everyone together. We also have to work at real cultural change within those open categories. Because male only sporting spaces, well, they're great for some guys, and they're terrible for the women they encounter. But they're also terrible for a lot of the boys and men. I would do an immediate overhaul, but I would emphasize restoring funding to community based sport. Kids should not be kept from participating in sport because they don't have the fees, or their parents can't drive them. We need more public funding for community based sports, the end of sex segregation, with girls and women only spaces that have trans inclusive boundaries on the basis of gender self determination. I think those would be my priorities.

### Sophia Nimphius

I believe a lot of challenging and humbling introspection is required by organisations (and institutions) for us to build a more inclusive and supportive environment not just for women and gender expansive people but for all people. I say this because diverse people will go to where they feel included and supported. If diverse people don't feel included or supported, they will not go to those places. So if our sporting teams, clubs, and administration are not diverse, that says something about the culture and whether independent of the statements a club might put out or a banner they may occasionally hang. If people show and truly demonstrate inclusion, diversity will follow and increase. We have to realise that inclusivity and supportive environments help everyone. There are unique glimpses of the benefit of learning from the extraordinary knowledge we are gaining from the growth of women's sporting competitions (increasing inclusivity) that provides reflection points for athletes in men's competitions, for example. Women that are in semi-professional or professional in sports that are also studying or working a second job because they, unfortunately, are not able to financially rely on sport gain additional skills, perspectives and although challenging timewise, enable an outlet that extends them from being “just an athlete” which can have positive benefits. Athletes in men's competitions, as noted in discussions of players' associations, are noting these positive attributes in the women's competitions after they move on from playing. They are further positioning and discussing their needs in reflection for a post-athletic career. We can all learn from each other if we are all in the room together. Right now, particularly in decision-making rooms, we simply are not.

### Nonhlanhla Mkumbuzi

Firstly, we need to acknowledge our biases. That means acknowledging that our life experiences are just that, ours. They are not universal and therefore how we perceive sport is not universal. That means acknowledging that these life experiences inform the way we view the world, sport included, and that view may not be the same as the next woman. We cannot improve what we do not measure and we cannot measure what we do not acknowledge. Acknowledgement of our current position towards inclusivity is the first step towards creating even more inclusive environments or improving on the ones we have. Secondly, we need to intentionally create infrastructure that purposefully includes women and gender diverse groups. They receive a bad rep, but quota systems work. If left to occur organically, inclusion of women and gender expansive people will not happen fast enough. We will definitely not achieve parity in our lifetimes and we will be singing the same tune 50 or 100 years from now. So quota systems in funding, hiring, publishing, media coverage, etc across the value chain would ensure that we ring fence resources for women and gender diverse groups. This is because in a world of scarce resources, when we are left to compete on an equal basis, women, children and those with disabilities will bear the brunt of the shortage. Sport is no different here. Lastly, we need representation of women and gender expansive people at all levels of sport, from the playing field to the boardroom. Decisions about issues that relate to women and gender diverse individuals should not be made without them in the room. Therefore, concerted efforts need to be made to ensure that we have representation at all levels of sport.

### Simone Fullagar

We could think about how to organise feminist scholars working across diverse areas and disciplines to connect in a rhizomatic way that can shape new thinking, research agendas and collaborations for different sport futures. I feel like we have been researching systemic forms of sexism for a long time and change is happening, so how do we consolidate and grow this momentum as a collective feminist force for change across the globe? An inclusive feminist politics that seeks transdisciplinary connections can produce new ways of thinking and expose the limitations of normative assumptions, exclusionary binaries and power relations that underpin knowledges informing sport communities and systems. Both science and social science researchers have identified how greater complexity and more nuance (and diverse voices) are needed to understand the sociopolitical conditions that shape gendered bodies in movement.

### Madeleine Pape

I mentioned above that shifting how IFs and sports doctors and scientists are thinking about eligibility criteria for the women's category is a long game. Building on Simone's response, I would add that this long game involves bringing not just feminist scholars on board but more cisgender women as well. There is a long tradition of feminist sports scholarship showing that organizing sport around an essentialist, heteropatriarchal binary is not good for any women, but especially those amongst us who are not privileged by able-bodiedness, whiteness, or the legacies of colonialism (32, 60, 61). Yet, if recent debates around trans athletes are anything to go by, we have a lot of work to do to make this understanding of the universal harms of the binary legible to the women who currently embrace this as the condition for their existence in elite sport. In coming full circle to the topic of athlete health and well-being, I believe and hope that it is possible to create a common cause with the voices that are currently advocating for the “female athlete health” agenda. The challenge is to bring them towards a feminist vision that includes all women and that does not reduce health to an essentialist binary, nor limit the right to health and safety to only those athletes that conform to and uphold the cisgender binary.

## Final thoughts and future agenda

This paper brings together the voices of six leading feminist sport and social science scholars, each of whom are involved in projects in which they are working across the disciplines in their efforts to improve the opportunities for women and gender diverse athletes in sport. This group brings together more than 120 years of living, breathing, sweating, fighting for more just, inclusive sporting models. The lived experiences, social and disciplinary positionings, and interpretations and applications of feminism vary, and yet there are also many similarities and points of intersection and synergy across the perspectives shared. We feel honoured to have heard about the contributors different research experiences, challenges and strategies, and their vision for the future of feminist innovation in sport studies. While their approaches differ, we believe there is much strength in the multiplicities of feminist approaches to sport studies. Too often feminist sport scholars are working in our disciplinary silos, this can be lonely, isolating, and frustrating. When we come together to listen and learn from one another, we can better articulate the similarities and differences across our disciplinary fields and our different positionings in sport and society. Furthermore, much of the scientific research on ‘female athlete health’ remains dominated by whiteness and voices of ‘experts’ from the Global North, and there is an urgent need to continue working towards more expansive approaches that value locality, and other ways of knowing and doing feminist research, advocacy and activism in sporting contexts. As noted above, we recognize that the dialogue offered herein is not representative of the full diversity of those working across disciplines and with sports organizations to create healthier, safer and more inclusive sporting environments for women and gender expansive persons. Yet, we hope this dialogue offers a starting point, a catalyst for ongoing conversations across the disciplines, as well as with feminist scholars from a wider array of geographical, gender and social positionings.

We conclude with some take home points from this dialogue, and strategies we can all work towards more innovative and collaborative feminist sport studies that have real life impacts for athletes around the world:

1. Resist biological essentialism (a lingering tendency to reduce understandings of gender to physiological sex differences, and to primarily focus on those sex differences). Unpack and resist the biological essentialist assumptions shaping ‘female athlete health’ research and practice, and recognise and incorporate *gendered* approaches to athlete health research. Although ‘female athlete health’ has become a common phrasing used in research, the media and in sports organizations, we recommend a move towards more gender inclusive language (i.e., people who menstruate; pregnant people, athletes who play in the women's category - not assuming that all who menstruate or become pregnant or play in the women's category are cisgender women).
2. Embrace sex/gender entanglement. Take up the entanglement of sex/gender in gendered approaches to athlete health research and practice for women and gender expansive people.
3. Intersectional, feminist, transdisciplinary. Move towards an explicitly intersectional feminist, transdisciplinary approach to athlete health and wellbeing. We can't understand gendered experiences without understanding the impact of white supremacy and racism, colonialism, ableism, classism, homophobia, transphobia, etc.
4. Listen and learn to overcome challenges. Recognise that working with sports organisations towards more gender responsive and inclusive approaches can be very challenging, and we could learn much from those who both are already working in this space, and those who have lived experience, as to strategies that do (or don't) work well.
5. Explore strategies to bridge academia and practice, and learn from those who are effectively navigating across these spaces. Opening more spaces for conversations as to the tensions, compromises, and approaches that work well, when doing feminist research with those from different disciplines, and in and with sports organizations.
6. Feminist movement. Establish a global network of feminist scholars, practitioners, and athletes from diverse social, cultural, disciplinary and geographical positioning to shape new thinking, research agendas, and collaborations for different and expansive sporting futures. This should include a ‘long game plan’ for locally specific sporting models that centre those who experience intersectional forms of oppression in order to drive feminist change within the sporting system.

## References

[1] Cowley E Olenick A McNulty K Ross E. ‘Invisible sportswomen’: the sex data gap in sport and exercise science research. Women in Sport and Phys Act J. (2021) 29(2):146–51. 10.1123/wspaj.2021-0028

[2] Mkumbuzi NS Chibhabha F Zondi PC. Out of sight, out of mind: the invisibility of female African athletes in sports and exercise medicine research. Br J Sports Med. (2021) 55(21):1183–4. 10.1136/bjsports-2021-104202 33975826

[3] Smith E McKay A Ackerman K Harris R Elliott-Sale K Stellingwerff T Methodology review: a protocol to audit the representation of female athletes in sports science and sports medicine research. Int J Sport Nutr Exerc Metab. (2021) 32(2):114–27. 10.1123/ijsnem.2021-0257 35168200

[4] Zhu J Reed J Van Spall HG. Underrepresentation of female athletes in sports research: considerations for cardiovascular health. Eur Heart J. (2021) 43(17):1609–11. 10.1093/eurheartj/ehab846 34904147

[5] Cain M. I was the fastest girl in America, until I joined Nike. (2019). Available: https://www.nytimes.com/2019/11/07/opinion/nike-running-mary-cain.html Google Scholar

[6] Fleshman L. I changed my body for sport. No girl should (2019). Available: https://www.nytimes.com/2019/11/16/opinion/girls-sports.html

[7] Parsons JL Coen SE Bekker S. Anterior cruciate ligament injury: towards a gendered environmental approach. Br J Sports Med. (2021) 55:984–90. 10.1136/bjsports-2020-103173 33692033

[8] Heather A Thorpe H Ogilvie M Sims S Beable S Milsom S Biological and socio-cultural factors have the potential to influence the health and performance of elite female athletes. Front Sport and Act Living. (2021) 8:Article 601420. 10.3389/fspor.2021.601420 PMC7932044 33681758

[9] Schofield K Thorpe H Sims S. Compartmentalised disciplines: why low energy availability research calls for transdisciplinary approaches. Perform Enhanc and Health. (2020) 8(2-3):100172. 10.1016/j.peh.2020.100172

[10] Schofield K Thorpe H Sims S. Feminist sociology confluences with sport science: insights, contradictions, and silences in interviewing women athletes about low energy availability. J Sport and Soc Issues. (2021) 46(3):223–46. 10.1177/01937235211012171

[11] Schofield K Thorpe H Sims S. ‘This is the next frontier’: power and knowledge in coaches ‘proactive’ approaches to sportswomen's Health. Sports Coaching Review. (2022) 11(3):324–45. 10.1080/21640629.2022.2060635

[12] Nimphius S. Exercise and sport science failing by design in understanding female athletes.”. Int J Sports Physiol Perform. (2019) 14(9):1157–8. 10.1123/ijspp.2019-0703 31553942

[13] Nimphius S McBride JM Rice PE Goodman-Capps CL Capps CR. Comparison of quadriceps and hamstring muscle activity during an isometric squat between strength-matched men and women. J Sports Sci and Med. (2019) 18(1):101–8. 30787657 PMC6370970

[14] Lawrence R Despres C. Futures of transdisciplinarity. Futures. (2004) 36:397–405. 10.1016/j.futures.2003.10.005

[15] Evans J Davies B. New directions, new questions? Social theory, education and embodiment. Sport Educ Soc. (2011) 16(3):263–78. 10.1080/13573322.2011.565960

[16] Markula P. Human bodies in motion: the performative intra-actions of materialization. Cultural Studies ⇔ Critical Methodol. (Forthcoming)

[17] Taylor C Ulmer J Hughes C. (Eds). Transdisciplinary feminist research: Innovations in theory, method and practice. Routledge: New York (2022).

[18] Thorpe H Clark M Brice J Sims S. The transdisciplinary health research apparatus: a baradian account of knowledge boundaries and beyond. Health. (2022) 26(3) 361–84. 10.1177/1363459320961429 33012195

[19] Thorpe H Brice J Rolleston A. Decolonizing sport science: high performance sport, indigenous cultures, and women's Rugby. Sociol Sport J. (2020) 37(2):73–84. 10.1123/ssj.2019-0098

[20] Pape M Bekker S Storr R Patel S Mitra P. Gender inclusive sport: a paradigm shift for research, policy, and practice. Int J Sport Policy and Polit. (Forthcoming).

[21] Bekker S Ahmed OH Bakare U Blake TA Brooks AM Davenport TE We need to talk about manels: the problem of implicit gender bias in sport and exercise medicine. Br J Sports Med. (2018) 52(20):1287–9. 10.1136/bjsports-2018-099084 29550755

[22] Bekker S Kolanyane-Kesupile KK. Fighting a system built to exclude queer (ing) bodies: an imperative for athlete wellbeing. In: Campbell N, Brady A, Tincknell-Smith A, editors. Developing and supporting athlete wellbeing (pp. 225–38). London: Routledge (2021).

[23] Bekker S Posbergh A. Safeguarding in sports settings: unpacking a conflicting identity. Qual Res Sport Exerc Health. (2022) 14(2):181–98. 10.1080/2159676X.2021.1920456

[24] Bekker S Storr R Posbergh A. Inclusion, fairness and non-discrimination in sport: a wider lens. Br J Sports Med. (2022) 56(19):1064–5. 10.1136/bjsports-2022-105926 35944970

[25] Fullagar S Pavlidis A. Feminist new materialist insights for sport management. In: Markula P Knoppers A, editors. Handbook on gender and diversity in sport management. Cheltenham and Camberley: Edward Elgar Publishing (2022).

[26] Forsdike K Fullagar S. A collaborative research agenda for women's Safety in sport: using the world café method to identify organisational responses to violence against women in sport. J Sport Management. (2021) 36(5):473–87. 10.1123/jsm.2020-0435

[27] Haraway D. Staying with the trouble: making kin in the chthulucene. Durham, NC: Duke University (2016).

[28] Epstein S. Inclusion: the politics of difference in medical research. Chicago: The University of Chicago Press (2007).

[29] Pape M. Co-production, multiplied: enactments of sex as a biological variable in US biomedicine. Soc Stud Sci*.* (2021) 51(3): 339–63. 10.1177/0306312720985939 33491581

[30] National Academies of Sciences. Engineering, and Medicine (NASEM). Improving the Health of Women in the United States: Workshop Summary. (2016) Washington, DC: The National Academies Press.

[31] Cahn S. Coming on strong: gender and sexuality in twentieth-century Women's Sport. Boston, MA: Harvard University Press (1994).

[32] Heywood L Dworkin S. Built to win: the female athlete as cultural icon. Minneapolis: University of Minnesota Press (2003).

[33] Hertzman C Boyce T. How experience gets under the skin to create gradients in developmental health. Annu Rev Public Health. (2010) 31:329–47. 10.1146/annurev.publhealth.012809.103538 20070189

[34] Mountjoy ML. (Ed). Female athlete handbook. International Olympic committee. John Wiley and Sons, Inc., Hoboken, New Jersey, Chapter 1 (2015).

[35] Pape M. Lost in translation? Beyond sex as a biological variable in animal research. Health Sociol Rev. (2022) 30(3):275–91. 10.1080/14461242.2021.1969981 34448683

[36] Karkazis K. The misuses of ‘biological sex’. Lancet. (2019) 394:1898–9. 10.1016/S0140-6736(19)32764-3 31982044

[37] Richardson SS. Sex contextualism. Philoso, Theory, and Pract in Biol. (2022) 14:2. 10.3998/ptpbio.2096

[38] Homan P. Structural sexism and health in the United States: a new perspective on health inequality and the gender system. Am Sociol Rev. (2019) 84(3):486–516. 10.1177/0003122419848723

[39] Nielsen MW Stefanick ML Peragine D Neilands TB Ioannidis J Pilote L Gender-related variables for health research. Biol Sex Diff. (2021) 12:23. 10.1186/s13293-021-00366-3 PMC7898259 33618769

[40] Dowling C. The frailty myth: women approaching physical equality. New York: Random House (2000).

[41] Williams P. The alchemy of race and rights. Cambridge, MA: Harvard University Press (1992).

[42] Mbembe A Meintjes L. Necropolitics. Public Culture. (2003) 15:11–40. 10.1215/08992363-15-1-11

[43] Puar J. Terrorist assemblages: homonationalism in queer times. Durham, NC: Duke University Press (2007).

[44] Haritaworn J Snorton CR. Transsexual necropolitics. In: Aizura AZ Stryker S, editors. The transgender studies reader 2. New York: Routledge (2013). p. 66–76.

[45] Karkazis K Jordan-Young R. The powers of testosterone: obscuring race and regional bias in the regulation of women athletes. Fem Form. (2018) 30(2):1–39. 10.1353/ff.2018.0017

[46] International Olympic Committee (IOC). IOC Framework on Fairness, Inclusion and Non-Discrimination on the Basis of Gender Identity and Sex Variations. (2021). Available at: https://stillmed.olympics.com/media/Documents/News/2021/11/IOC-Framework-Fairness-Inclusion-Non-discrimination-2021.pdf

[47] World Medical Association (WMA). WMA Declaration on principles of health care in sports medicine, 2021. (2021) Available: https://www.wma.net/policies-post/wma-declaration-on-principles-of-health-care-for-sports-medicine/15584153

[48] Pigozzi F Bigard X Steinacker J Wolfarth B Badtieva V Schneider C Joint position statement of the international federation of sports medicine (FIMS) and European federation of sports medicine associations (EFSMA) on the IOC framework on fairness, inclusion and nondiscrimination based on gender identity and sex variations. BMJ Open Sport and Exer Med. (2022) 8:e001273. 10.1136/bmjsem-2021-001273 PMC8739444 35127133

[49] Karkazis K Carpenter M. Impossible ‘choices’: the inherent Harms of regulating Women's Testosterone in sport. J Bioeth Inq. (2018) 15(4):579–87. 10.1007/s11673-018-9876-3 30117064

[50] Karkazis K Jordan-Young R Davis G Camporesi S. Out of bounds? A critique of the new policies on hyperandrogenism in elite female athletes. Am J Bioeth. (2012) 12(7):3–16. 10.1080/15265161.2012.680533 22694023 PMC5152729

[51] Fédération Internationale de Natation (FINA). Policy on eligibility for the men's and women's categories. (2022) Available at: https://resources.fina.org/fina/document/2022/06/19/525de003-51f4-47d3-8d5a-716dac5f77c7/FINA-INCLUSION-POLICY-AND-APPENDICES-FINAL-.pdf

[52] Pieper LP. Sex testing: gender policing in Women's Sport. Urbana Champaign, IL: University of Illinois Press (2016).

[53] Davis G. Contesting intersex: the dubious diagnosis. New York: New York University Press (2015).

[54] Henne K Pape M. Dilemmas of gender and global sports governance: an invitation to southern theory. Sociol Sport J. (2018) 35(3):216–25. 10.1123/ssj.2017-0150

[55] Human Rights Watch. “They’re chasing us away from sport” Human rights violations in sex testing of elite women athletes. (2020) Available at: https://www.hrw.org/report/2020/12/04/theyre-chasing-us-away-sport/human-rights-violations-sex-testing-elite-women

[56] Acosta RV Carpenter LJ. *Women in Intercollegiate Sport: A Longitudinal, National Study. Thirty-Five Year Update, 1977-2012*. Acosta-Carpenter (2012).

[57] Connell R. Gender and Power: society, the Person and Sexual Politics. Allen and Unwin. (1987).

[58] Courville J Vézina N Messing K. Comparison of the work activity of two mechanics: a woman and a man. Int J Ind Ergon. (1991) 7:163–74. 10.1016/0169-8141(91)90045-N

[59] Leahy T Pretty G Tenenbaum G. Prevalence of sexual abuse in organized competitive sport in Australia. J Sexual Aggress. (2002) 8(2):16–36. 10.1080/13552600208413337

[60] McDonagh EL Pappano L. Playing with the boys: why separate is not equal in sports. Oxford: Oxford University Press (2008).

[61] Travers A. The sport nexus and gender injustice. Studies in Social Justice. (2008) 2(1):79–101. 10.26522/ssj.v2i1.969

